# Early Marrow Microenvironment Immune Patterns After Hematopoietic Stem Cell Transplant in Pediatric Acute Lymphoblastic Leukemia Are Associated with Later Development of Chronic GvHD and Relapse

**DOI:** 10.3390/ijms27052338

**Published:** 2026-03-02

**Authors:** Catherine M. Njeru, Bernard Ng, Sayeh Abdossamadi, Alima Suleimenova, Carmen Dolores De Luca, Vaishnavi Parthasarathy, Laura M. Sly, Gregor S. D. Reid, Chia Huan Ng, Kirk R. Schultz

**Affiliations:** 1 Michael Cuccione Childhood Cancer Research Program, British Columbia Children’s Hospital Research Institute, University of British Columbia, Vancouver, BC V5Z 4H4, Canada; njerucathy@gmail.com (C.M.N.); alima.suleimenova@bcchr.ca (A.S.); carmen.deluca@bcchr.ca (C.D.D.L.);; 2 Department of Statistics, Centre for Molecular Medicine and Therapeutics, British Columbia Children’s Hospital, University of British Columbia, Vancouver, BC V5Z 4H4, Canada; 3 Department of Pediatrics, British Columbia Children’s Hospital Research Institute, University of British Columbia, 950 West 28th Ave, Rm 3102, Vancouver, BC V5Z 4H4, Canada; 4 Department of Hematology/Oncology, Cell and Gene Therapy, Istituto di Ricovero e Cura a Carattere Scientifico (IRCCS), Bambino Gesù Children’s Hospital, 00165 Rome, Italy; 5 Allen Institute for Immunology, Seattle, WA 98109, USA; 6 Starship Blood and Cancer Centre, Starship Children’s Hospital, Auckland 1142, New Zealand

**Keywords:** chronic GvHD, modeling immune patterns, graft-versus-leukemia, hematopoietic cell transplantation

## Abstract

Hematopoietic stem cell transplant (HSCT) is a curative therapy for acute lymphoblastic leukemia (ALL), but its success is limited by chronic graft-versus-host disease (cGvHD) and disease relapse. A central challenge is uncoupling the graft-versus-leukemia (GvL) effect from cGvHD. Early changes in the bone marrow microenvironment following HSCT may offer a predictive window into these divergent outcomes. We conducted a retrospective, single-center, exploratory study on 14 pediatric ALL HSCT patients. Applying single-cell antibody-sequencing (AbSeq) on archived bone marrow aspirates collected 60–100 days post-HSCT, we evaluated immune patterns associated with the development of cGvHD or ALL relapse after day 114. cGvHD after day 114 was associated with upregulation of the endoplasmic reticulum (ER) stress transcription factor *XBP1* in transitional B cell and IgM memory B cell populations, a mincle^high^PD1^−^ neutrophil population, and exhausted *LAG3*^+^ effector memory T cells (T_EM_). ALL relapse after day 114 was associated with higher *CD22*, *CD24*, and *ARG1* expression in M(IL-4)-like macrophages and exhausted *TIGIT*^+^ T_EM_. Results from this exploratory study suggest that marrow immune signatures of B cell ER stress preceding later development of cGvHD and macrophage-mediated immune evasion preceding relapse may potentially be early biomarkers for separating GvL from cGvHD in ALL HSCT. Validation with larger cohorts is warranted.

## 1. Introduction

Hematopoietic stem cell transplant (HSCT) is an established therapy that induces immune-mediated graft-versus-leukemia (GvL) effects, which contribute to durable remission in acute leukemia [1,2]. The GvL effect is primarily driven by donor T cells that recognize and eliminate recipient leukemia cells by targeting minor histocompatibility and leukemia-associated antigens. This adaptive response is critically supported by innate immune players like NK cells, which kill leukemia cells that lack inhibitory “self” signals, while macrophages and B cells amplify the T cell attack through antigen presentation [3,4]. HSCT is often employed as a rescue therapy for refractory acute lymphoblastic leukemia (ALL) following unsuccessful conventional chemotherapy or targeted immune therapies [5]. However, the success of HSCT is limited by chronic graft versus host disease (cGvHD), an off-target complication with multisystem effects that can last for many years, leading to morbidity and mortality [6,7].

cGvHD has a complex pathogenesis that involves both the innate and adaptive immune systems and comprises three major components or phases, characterized by tissue injury, chronic inflammation, dysregulated immunity, and aberrant tissue repair with fibrosis [8,9]. cGvHD is driven by the dysregulation of B cell homeostasis, leading to a loss of tolerance. This allows for the survival and activation of pathogenic B cells, which, in collaboration with alloreactive T cells, ultimately result in the inflammation and fibrosis characteristic of the disease. Natural killer (NK) cells, NKT cells, monocytes, and macrophages also contribute to immune system dysregulation, while regulatory populations of T cells, B cells, myeloid cells (monocytes, macrophages, myeloid-derived suppressor cells), and NK cells can ameliorate the process [9]. Our immune profiling of cGvHD has revealed that rare immune cell populations may be important in the biology of the disease. For example, we have identified unique T_reg_, NK_reg_, and transitional B cell populations that correlate strongly with cGvHD [10]. More recently, we found that there are distinct biological subtypes of cGvHD in peripheral blood as characterized by unique patterns of cell subsets [11].

cGvHD is potentially associated with the GvL effect and reduces the risk of relapse [8], although this interaction is not yet fully understood in humans [12]. Optimizing the GvL effect while preventing cGvHD remains a primary challenge in HSCT. As a deeper understanding of early immune changes in the marrow microenvironment may help achieve this goal, in this exploratory study with 14 ALL patients, we utilize antibody–oligonucleotide conjugates (AbSeq), which leverage oligonucleotide sequencing, to investigate early transcriptome changes in days 60–100 post HSCT marrow microenvironment that are associated with the development of cGvHD and ALL relapses with sustained GvL effect.

## 2. Results

### 2.1. Cell Populations Identified in Day 100 Marrow After HSCT

**Figure 1:**
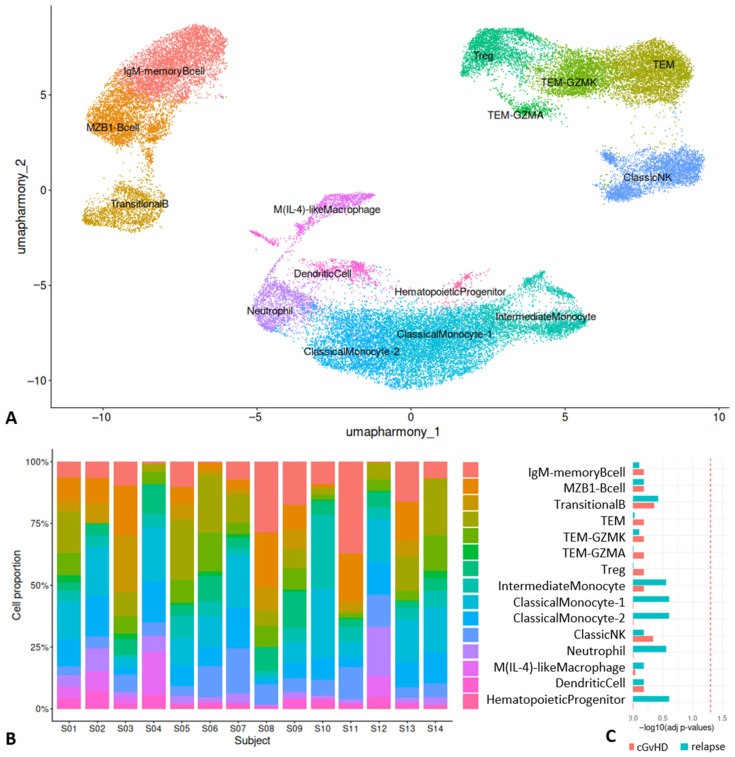
Cell types in marrow. (**A**) UMAP of cell clusters with their annotated cell types. (**B**) Cell type proportion within each patient. (**C**) Cell type proportions were contrasted between patients who later developed cGvHD vs. no cGvHD, as well as between patients who later developed ALL relapse vs. no relapse. −log10 of FDR-adjusted *p*-values displayed. Red line corresponds to a threshold of −log10(0.05).

We identified a number of immune populations in marrow between days 60 and 100 (Figure 1A), including B cell populations, such as marginal zone (MZ) B cells (based on markers such as CD10^+^, CD19^+^, CD24^+^, *MZB1^+^*), transitional B cells (CD10^+^, CD19^+^, CD24^+^, CD27^−^, IgD^−^, IgM^−^), and IgM memory B cells (CD10^+^, CD19^+^, CD24^+^, CD21^+^, IgD^+^, CD27^−^, IgM^+^). T cell populations included effector memory T (T_EM_) cells (CD3^+^, CD8^+^, CD45RA^−^), Granzyme K expressing (GMZK) T_EM_ (CD3^+^, CD8^+^, CD27^+^, C45RA^−^, *GZMK^+^*), Granzyme A expressing (GMZA) T_EM_ (CD3^+^, CD8^+^, CD45RA^−^, CD27^+^, CD70^+^, *GZMA^+^*), and regulatory T (T_reg_) cells (CD3^+^, CD4^+^, *FOXP3^+^*). NK cells included classic cytolytic natural killer (NK) cells (CD56^+^, CD16^+^). Myeloid populations included M(IL-4)-like macrophages (*ARG1^+^*, *ALAS2^+^*, CD36^+^, *TGFB3^+^*, *CD63^+^*, *IL1B^+^*, *STAT6^+^*), neutrophils (*ELANE^+^*, *RNASE2^+^*), dendritic cells (CD11c^+^, *FCER1A^+^, CLEC1-A^+^),* classic monocyte populations 1 and 2 (CD14^+^, CD11c^+^, CD16^+^), an intermediate monocyte population (CD14^+^, CD16^+^, CD11c^+^), and hematopoietic progenitor cell population (CD4^+^, CD27^+^, CD16^+^). These cell types were present in all patients’ marrow (Figure 1B), and we observed no differences in cell type proportions between cGvHD vs. non-cGvHD nor ALL relapse vs. no relapse (Figure 1C), with false discovery rate (FDR) correction for all combinations of cell types and group contrasts.

### 2.2. Day 60–100 Protein and Gene Expression Patterns Associated with Later Chronic GvHD

**Figure 2:**
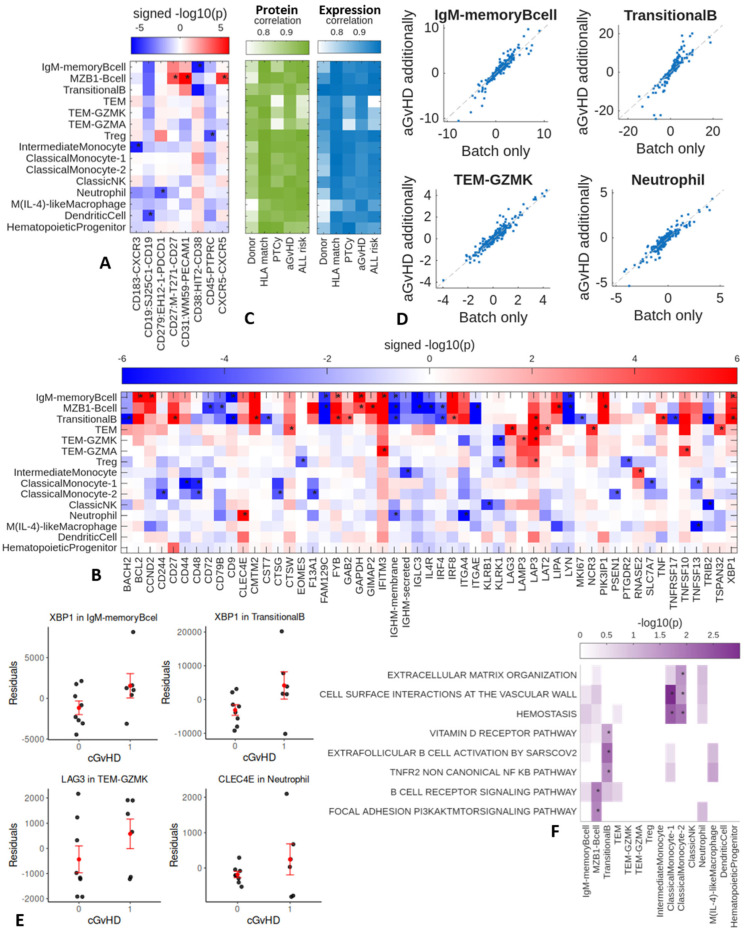
cGvHD differential proteins and genes. (**A**) Heatmap of differential proteins and (**B**) differentially expressed genes in cGvHD. * indicates proteins and genes with NEBULA *p*-value passing FDR correction, pseudobulk *p*-value < 0.05, and estimated effect directions matching between NEBULA and pseudobulk analysis. The displayed signed −log(*p*) corresponds to *p*-values estimated with NEBULA. (**C**) We repeated the NEBULA analysis with each clinical factor additionally included as a confounder in the model. Displayed are correlations between the signed −log10(*p*) across all proteins/genes with and without accounting for a given clinical factor. (**D**) Scatterplots of signed −log10(*p*) with and without modeling aGvHD as a confounder in NEBULA for cell types with more DEGs. Each dot corresponds to a gene. (**E**) Dot plots of pseudobulk expression of exemplar DEGs after regressing out batch and relapse. (**F**) We evaluated the overlap between DEGs and GO genesets using Fisher’s exact test. −log10(*p*) of genesets with enrichment *p* < 0.05 (indicated by *) for at least one cell type displayed.

Evaluating differential cell surface protein expression in patients who developed cGvHD compared to those who did not, we observed higher expression of CD27, CD31, and CXCR5 in the MZB cell population for patients who later developed cGvHD (Figure 2A). The IgM memory B cell population showed lower cell surface expression of CD38, and the intermediate monocyte cell population showed lower expression of CD183 (CXCR3). Some clinical factors (Table 1 and Table 2), such as donor source, HLA matching, use of PTCy, previous acute GvHD (aGvHD), and ALL risk, could influence the associations between proteins and cGvHD. Therefore, we repeated the differential protein analysis with each of these clinical factors modeled as a confounder. We then compared the signed *p*-values across proteins with and without accounting for these clinical factors. The high correlation (Figure 2C) between the signed *p*-values with and without accounting for clinical factors indicates that these factors only mildly affect the prioritization of the proteins.

Evaluation of differentially expressed genes (DEG) in cGvHD (Figure 2B) revealed higher expression of *GAPDH* in the MZB cell and the IgM memory B cell populations, and higher expression of *XBP1* in both the transitional B cell and IgM memory B cell populations (Figure 2E). The IgM memory B cell population also showed higher expression of *IFITM3, BCL2, CCND2,* and *FYB* and lower expression of *CD9*, *FAM129C*, and *IgHM*. The MZB cell population showed higher expression of *PIK3IP1*, *LIPA*, and *GIMAP2* and lower expression of *CD272*, *CD79B*, *FAM129C*, *IgHM*, *IL4R*, *IRF4*, *IGLC3*, and *MKI67*. The transitional B cell population showed higher expression of *CD27*, *CMTM2*, *FYB*, *GAB2*, *IRF8*, *LAP3*, and *TNF* and lower expression of *BACH2*, *CD9*, *IRF4*, *MKI67*, *TNF5F17*, and *TRIB2*. In the T cell populations, the T_EM_ population showed higher expression of *LAP3*, *LAT2*, *NCR3*, *LAG3*, *TSPAN32* and *CTSW*. The Granzyme K^+^ T_EM_ population showed higher expression of *LAMP3* and *LAP3,* and the T_reg_ population showed higher expression of *LAP3*. Thus, all three of the T cell populations (T_reg_, Granzyme K^+^ T_EM_, and T_EM_) showed higher expression of *LAP3*. A fourth T cell population (Granzyme A^+^ T_EM_) showed higher expression of *IFITM3* and *TNFSF10*, but not *LAP3*. The NK cell population showed lower expression of *KLRB1* and *TRIB2*. The Classic Monocyte 2 population showed lower expression of *CD48*, *CD244*, *CTSG*, *F13A1*, and *PSEN1*. The intermediate monocyte population showed higher expression of *RNASE2*. The neutrophil population showed higher expression of macrophage-inducible C-type lectin (Mincle, *CLEC4E*), and lower expression of *CD279* (*CD1PD1*) and *ITGA4*. Considering that some clinical factors could influence the associations between genes and cGvHD, we repeated the differential expression analysis with clinical factors additionally modeled as confounders and compared the signed *p*-values across genes with and without these additional confounders. The high correlation between the signed *p*-values with and without accounting for clinical factors indicates that these factors only mildly affect the gene prioritization (Figure 2C,D). To aid interpretation, we evaluated the overlap between the DEGs and GO genesets using Fisher’s exact test. We found enrichment of the *TNFR2* non-canonical *NF-κB* pathway and vitamin D signaling for transitional B cells, and as expected, enrichment of B cell signaling pathways for MZB cells (Figure 2F).

### 2.3. Day 60–100 Protein and Gene Expression Patterns Associated with Later ALL Relapses

**Figure 3:**
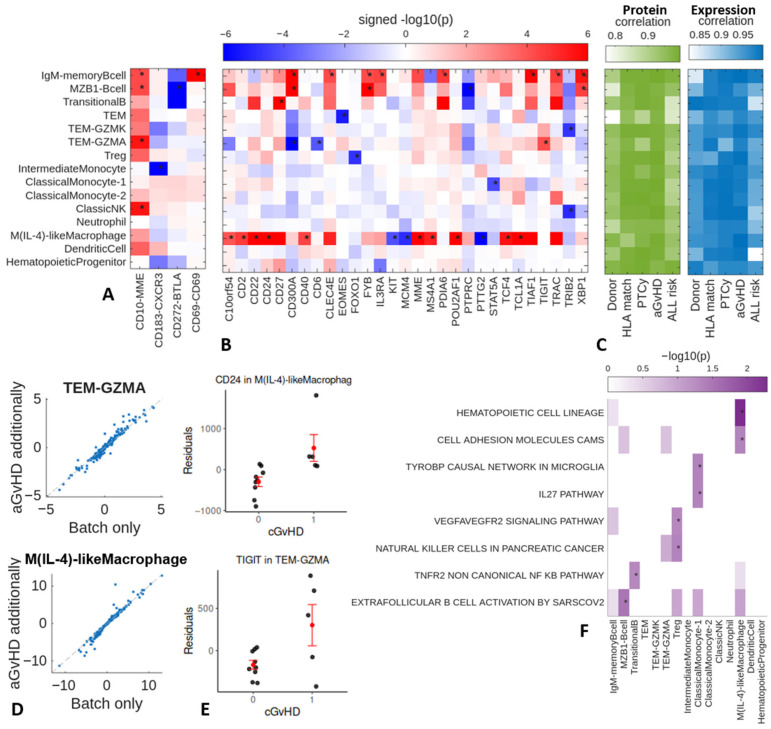
ALL relapse differential proteins and genes. (**A**) Heatmap of differential proteins and (**B**) differentially expressed genes in relapse. * indicates proteins and genes with NEBULA *p*-value passing FDR correction, pseudobulk *p*-value < 0.05, and estimated effect directions matching between NEBULA and pseudobulk analysis. The displayed signed −log(*p*) corresponds to *p*-values estimated with NEBULA. (**C**) We repeated the NEBULA analysis with each clinical factor additionally included as a confounder in the model. Displayed are correlations between the signed −log10(*p*) across all proteins/genes with and without accounting for a given clinical factor. (**D**) Scatterplots of signed −log10(*p*) with and without modeling aGvHD as a confounder in NEBULA for cell types with more DEGs. Each dot corresponds to a gene. (**E**) Dot plots of pseudobulk expression of exemplar DEGs after regressing out batch and cGvHD. (**F**) We evaluated the overlap between DEGs and GO genesets using Fisher’s exact test. −log10(*p*) of genesets with enrichment *p* < 0.05 (indicated by *) for at least one cell type displayed.

Patients who had a future ALL relapse compared to those who did not, showed higher expression of CD10 on NK and Granzyme A^+^ T_EM_. In the B cell populations, MZB cells showed higher expression of CD10 and lower expression of CD272 (*BTLA*), and IgM memory B cells expressed higher levels of CD10 and CD69 (Figure 3A). The intermediate monocytes showed lower expression of the cell surface protein CD183 (CXCR3). Repeating the differential protein analysis with clinical factors additionally modeled as confounders resulted in signed *p*-values that highly correlated with those found without accounting for these clinical factors (Figure 3C), indicating these factors minimally affect the prioritization of the proteins.

Evaluating differential expression in ALL relapses (Figure 3B), the dominant pattern is higher expression of *CD22* (Figure 3E), *CD24*, *CD2*, *CD40*, *MME*, *MS4A1*, *POU2AF1*, *TCF4*, and *TCL1A* in M(IL-4)-like macrophages [13]. In addition, there were atypical T_EM_ populations, including a Granzyme A^+^ T_EM_ population, that showed higher expression of the exhaustion marker *TIGIT*, and lower expression of *CD300A* and *CD6*. Granzyme K^+^ T_EM_ showed lower expression of *TRIB2*, and T_reg_ cells showed lower expression of *FOXO1*. Furthermore, two of the B cell populations showed higher expression in *CD300A*, *FYB*, and *XBP1*. Repeating the differential expression analysis with clinical factors additionally modeled as confounders minimally affects the prioritization of the genes (Figure 3C,D). The DEGs in M(IL-4)-like macrophages were enriched for cell adhesion molecules (CAMs) and hematopoietic cell lineage (Figure 3F). Note that with only AbSeq measurements at day 100, we could not differentiate whether the B cell gene expression changes reflected early minimal disease B-ALL leading to a later relapse or corresponded to a non-malignant pattern.

## 3. Discussion

Although numerous marrow microenvironment studies have been performed, this study is the first that we are aware of to evaluate the marrow microenvironment before the relapse of ALL and development of cGvHD in children who underwent HSCT. We found two distinct patterns at day 100 that delineated the divergence between patients who later developed cGvHD and those who later developed relapse. cGvHD had a predominance of dysfunctional B cell populations and exhausted effector memory T cells. In contrast, ALL relapse was dominated by expression changes in M(IL-4)-like macrophages and a different T cell exhaustion pattern.

B cells are an important immune cell population in the development of cGvHD [14,15,16,17]. B cell-directed agents, such as the BTK inhibitor ibrutinib, are well established as FDA-approved agents to treat cGvHD [18]. While there have been numerous studies of dysfunctional B cell types in cGvHD, these studies have focused on peripheral blood B cells. The association between B cell dysfunction in the marrow microenvironment and the development of cGvHD is poorly understood. Here, we identified two distinct transitional B cell populations and a mixed population of IgM memory B cells that are associated with the subsequent development of cGvHD. A predominant finding is the positive association between cGvHD and the upregulation of *XBP1*, a master transcription factor that, in its spliced form (*XBP1*s), is essential for the terminal differentiation of B cells into high-rate antibody-secreting plasma cells [19]. Upregulation of *XBP1* is not merely a marker of differentiation, but also a direct indicator of cellular ER stress. We also saw an increase in *LAP3* expression in both B and T cell populations. While *LAP3* has been implicated in type I interferon production in T cells [20], the role of *LAP3* in T and B cell function in the cGvHD microenvironment is not clear.

The patterns seen in the atypical T_EM_ populations in patients who later developed ALL relapse included a granzyme K^+^ T_EM_ cell population that has a number of roles in immunity [21,22,23]. We observed that Granzyme K^+^ T cells overexpressed *LAMP3* and *LAP3* in patients who would later develop cGvHD, but saw a different pattern in those who progressed to ALL relapse with lower expression of *TRIB2*. Granzyme K^+^ Tc cells have a well-established role in autoimmunity [22,24] and exhibit a non-classical cytotoxic phenotype, with decreased cytotoxicity but heightened proinflammatory potential.

Granzyme A^+^ T_EM_ in cGvHD showed higher expression in *IFITM3* and *TMFSF10*, whereas the same population showed higher expression in *TIGIT* and lower expression in *CD300A* and *CD6* in those who later developed ALL relapse. This population is poorly understood, but Granzyme A^+^ Th cells have been associated with the development of acute GvHD in murine models [25]. *IFITM3* expression in marrow T cells has been associated with marrow T cell immune activity [26], and *TRAIL* is associated with autoimmunity in a number of settings [27,28].

M(IL-4)-like (ARG1^+^) tumor macrophages share features with other tumor-associated macrophages (TAMs), which are central players in suppression of the immune response against malignant cells [13]. In this analysis, M(IL-4)-like macrophages showed higher expression of *CD22* (Siglec-2) and *CD24* in patients who relapsed. *CD24* and *CD22* expression on tissue resident macrophages in the liver and lungs has been associated with anti-inflammatory effects and can be involved in immunologically silent clearance by efferocytosis in M(IL-4) macrophages [29,30,31,32,33]. Higher expression of *CD24* in hepatic tissue-resident liver macrophage populations can be induced by DAMPs during liver injury and suppresses inflammation signaling mainly through *CD24*-Siglec-G interaction [33]. The pro-tumor effects of M(IL-4)-like macrophages, formerly called M2-like TAMs, can be divided into three functions, including angiogenesis, immunosuppression, and tumor progression. MM(IL-4)-like TAMs secrete a number of growth factors, including *VEGF*, platelet-derived growth factor, epidermal growth factor, and TGF-β, as well as matrix metalloproteinases (*MMP-2*, *MMP-9*), and the cytokines TNF-α, IL-1β, and IL-8. Immune suppression by MM(IL-4)-like TAMs results in metastasis and inhibition of T cell and natural killer cells [31,32,33]. Thus, the presence of M(IL-4)-like macrophages in the marrow microenvironment would be consistent with previously identified tissue resident anti-inflammatory macrophage populations, though co-expression of *CD24* and *CD22* on tissue resident macrophages has not previously been described and should be an area of future investigation. The M(IL-4)-like macrophage population also showed higher expression in MME or *MMP12* in relapsed patients. Blockade of MMP12 can inhibit M(IL-4) macrophage activation [34]. Moreover, *MMP12* expression has been associated with increased tumor infiltration by FOXP3^+^T_reg_ and poor response to therapy in hepatocellular carcinoma [35]. The role of MMP12^+^ M(IL-4)-like macrophages in this setting is not as clear.

A macrophage-inducible C-type lectin (*CLEC4E*) Mincle^high^ neutrophil population was found to be associated with cGvHD. It is well established that macrophages play an important role in the fibrotic process, which is a major component of cGvHD pathology [36]. Accordingly, Mincle^high^ neutrophils appear to exhibit remarkable pro-inflammatory and pro-fibrotic functions [37]. The role of Mincle^high^ neutrophils has not previously been identified in peripheral blood studies of patients who develop cGvHD and may exist only within the marrow microenvironment.

Interestingly, we observed different T cell exhaustion marker patterns expressed by T_EM_ in cGvHD compared to T_EM_ associated with future ALL relapse. Higher *TIGIT* expression in Granzyme A^+^ T_EM_ was seen in ALL relapse patients, whereas higher *LAG3* expression in Granzyme K^+^ T_EM_ was observed in the marrow of those who later developed cGvHD. CTLA-4 and PD-1 are considered first wave checkpoint molecules, whereas LAG-3 and TIGIT appear to represent a second wave in immune regulation [38]. In addition, it appears that LAG-3 and TIGIT have different regulatory functions in T and NK cell function [39,40,41,42]. To date, checkpoint inhibitors targeting the first wave checkpoint molecules have been used in a number of studies post-HSCT. Unfortunately, among patients receiving checkpoint inhibitors before allo-HSCT, 56% developed aGvHD and 29% developed cGvHD. That same patient cohort had reported 20 deaths, 60% of which were GvHD-related [43]. Targeting TIGIT as a secondary checkpoint may have less toxicity and/or be a better target based on marrow microenvironment profiling in this study. Outside the HSCT setting, TIGIT blockade appears to repolarize TIGIT^+^M(IL-4)-like macrophages to a pro-inflammatory activation phenotype, inducing phagocytosis in AML-associated macrophages [44]. This is supported by the preclinical finding that mice treated with TIGIT-CD155 blockade (via anti-CD155 antibody) showed an improvement in EFS without GVHD exacerbation [45].

Targeting checkpoint interactions such as the CD24/Siglec-10 or even CD22 pathways also has the potential to restore a potent GvL effect [46] without increasing the development of GvHD. While checkpoint inhibition of the CD24/Siglec-10 pathway has previously been reported to depend on Siglec-10 expression on TAMs, it is possible that blockade of CD24 on CD24^+^ TAMs might decrease T cell exhaustion as well [47,48]. There is little known regarding a possible role of CD22 on TAMs as checkpoint inhibitors. The lower CD272 (B and T lymphocyte attenuator, *BTLA*) expression in B cells in relapsing patients was interesting, as BTLA can induce CD8^+^ T cell exhaustion and does so cooperatively with PD-1 [49,50]. Moreover, BTLA blockade can increase the antitumor efficacy of anti-PD-L1 therapy, and CD272 expression has been associated with B cell activation and may increase their expansion and differentiation into plasma blasts [51].

## 4. Materials and Methods

### 4.1. Patient Population and Selection

The archived bone marrow mononuclear cells and biopsy specimens analyzed were from pediatric patients with ALL who underwent HSCT between 2014 and 2021 at British Columbia Children’s Hospital (BCCH) and consented for biobanking. These samples were obtained from the BCCH biobank. Consenting to biobank and release for research purposes was approved by the local research ethics board at BC Children’s Hospital. The median age of the patients at transplant was 13 years. The most common conditioning regimen was TBI/Cyclophosphamide. Only one patient had a non-TBI regimen, using a combination of Fludarabine, Thiotepa, and Treosulfan (Modified FORUM protocol). Four out of 14 patients had post-transplant cyclophosphamide as part of their conditioning regimen. A majority (11/14) of patients received Tacrolimus and Mycophenolate Mofetil as part of their GvHD prophylaxis.

Clinical data were acquired through a retrospective review of online chart records on Cerner Power Chart. Patients were deidentified and assigned study IDs to preserve their anonymity. Cases where the development of cGvHD or leukemia relapse was uncertain were brought up for consensus discussion and attribution amongst clinical co-investigators. Fourteen samples were obtained for the study. Day 100 sample selection was restricted to those who had developed either chronic GvHD or pre-B-ALL relapse greater than 114 days post HSCT. The samples were distributed as follows: 1 patient was both ALL-relapse positive and cGvHD positive (Relapse^+^/cGvHD^+^); 4 patients were ALL-relapse positive and cGvHD negative (Relapse^+^/cGvHD^−^); 5 patients were ALL-relapse negative and cGvHD positive (Relapse^−^/cGvHD^+^); and 4 patients were ALL-relapse negative and cGvHD negative (Relapse^−^/cGvHD^−^). This resulted in a total of 5 B-ALL relapse patients (4 with a Relapse^+^/cGvHD^−^ and 1 with Relapse^+^/cGvHD^+^) compared to a total of 9 that had no relapse (5 Relapse^−^/cGvHD^+^ and 4 Relapses^−^/cGvHD^−^) for analysis. For evaluation of cGvHD patterns, 6 patients with cGvHD (5 with ALL Relapse^−^/cGvHD^+^ and 1 with ALL Relapse^+^/cGvHD^+^) were compared to 8 who were cGvHD negative (4 with ALL Relapse^+^/cGvHD^−^ and 4 with ALL Relapse^−^/cGvHD^−^).

Diagnosis of cGvHD was defined as per the 2014 NIH cGvHD diagnostic criteria [52], after confirmation by blinded observers for cases with an onset between days 114 and 365 post HSCT. A lack of marrow relapse between days 114 and 365 was considered a patient positive for GvL, and a patient who had an ALL relapse in the time period was considered negative for GvL. Marrow relapse was defined as reappearance of >5% ALL leukemic cells in the marrow after remission had been achieved (Table 1 and Table 2).

### 4.2. Targeted Single-Cell Transcriptome and Surface Profiling Using AbSeq

Frozen bone marrow mononuclear cells were thawed and washed in DPBS supplemented with 2% FBS. Cells were stained with anti-human CD45-APC (eBioscience, San Diego, CA, USA, clone 2D1, Cat # 17-9459-41) and 7-AAD (BioLegend, San Diego, CA, USA, Cat # 420404), and live CD45+ cells were sorted using a BD FACSAria IIu Cell Sorter (BD Biosciences, San Jose, CA, USA) for downstream AbSeq analysis. Sorted cells were labeled with a panel of oligonucleotide-conjugated antibodies (AbSeq, BD Biosciences) to quantify surface protein expression. The panel included the BD AbSeq Immune Discovering Panel (Cat # 625970), which contains 30 surface antibodies, Table S1) along with 11 individual Abseq antibodies: Hu CD5, clone UCHT2, Cat # 940038; Hu CD10, clone HI10A, Cat # 940045; Hu CD13, clone WM15, Cat # 940044; Hu CD21, clone B-LY4, Cat # 940048; Hu CD24, clone ML5, 940028; Hu CD31, clone WM59, Cat # 940254; Hu CD38, clone HIT2, Cat # 940013; Hu CD45, clone HI30, Cat # 940002; Hu CD69, clone FN50, Cat # 940019; Hu CD 335, clone 9E2/NKp46, Cat # 940064; Hu CD337, clone P30-15, Cat # 940291, all from BD Biosciences, San Jose, CA, USA. For multiplex analysis, samples were tagged using the BD^®^ Human Single-Cell Multiplexing kit (BD Biosciences, Cat # 633781) and pooled prior to capture. Labeled cells were loaded onto the BD Rhapsody^TM^ Single-Cell Analysis System (BD Biosciences), where single cells were captured along with AbSeq oligonucleotides.

Libraries were prepared following the BD Rhapsody^TM^ Targeted mRNA and AbSeq kit protocol (BD Biosciences, Cat # 633771), including reverse transcription, cDNA amplification, and library indexing. Library concentrations were measured using a Qubit Fluorometer (ThermoFisher Scientific, Waltham, MA, USA), and quality control of sequencing libraries was performed using an Agilent 2100 Bioanalyzer (Agilent Technologies, Santa Clara, CA, USA) to confirm fragment size distribution. Using the BD Rhapsody Sequencing Calculator, the appropriate amount of each library type was determined to achieve the desired read depth per cell. Fourteen samples were processed across three DB Rhapsody cartridges, after which all libraries were combined into a single library with a total volume of 200 μL for sequencing. Sequencing was performed at The Centre for Applied Genomics (TCAG) at The Hospital for Sick Children (Toronto, ON, Canada) on an Illumina NovaSeq 6000 (Illumina, San Diego, CA, USA) using an S4 flow cell, achieving a total sequencing depth of approximately 4.74 million reads (+1% Phix).

Sequencing data were processed using BD Rhapsody Sequencing Analysis Pipeline to quality-check the raw sequencing reads, identify and correct cell barcodes and unique molecular identifiers (UMIs), align reads to the reference transcriptome or known oligo sequences, and generate raw expression matrices for mRNA, SMK (Sample Multiplexing Kit, Cat # 633781, BD Biosciences, San Jose, CA, USA), and AbSeq libraries. Secondary analysis was then conducted on the Seven Bridges cloud platform, including normalization, alignment, and integration of transcriptomic and proteomic data, preparing the data set for visualization and statistical analyses. We measured expression of 397 genes and 41 proteins from 50,743 cells in total. As quality control, we removed multiplets as well as cells with Unique Molecular Identifier (UMI) counts < 50, number of genes < 35, and log10(number of genes per UMI) < 0.55.

### 4.3. Statistical Analyses

Using SEURAT with its default parameters [53], we first normalized the gene expression count matrix with “NormalizeData”. We then identified the highly variable genes using “FindVariableFeatures” and standardized the data using “ScaleData”. Subsequently, we extracted 30 principal components (PC) using “RunPCA” and integrated the gene expression data across subjects using “IntegrateLayers” (with Harmony as the integration method). Lastly, we generated a nearest neighbor graph using “FindNeighbors” and clustered the cells using “FindClusters”, resulting in 15 cell clusters. We visualized the clusters using UMAP. To determine the cell type of each cluster, we contrasted the gene expression values of cells within each cluster against all other clusters using “FindAllMarkers” to find the most differentially expressed genes for each cluster. Additionally, we normalized the protein count matrix with “NormalizeData” and applied “FindAllMarkers”. Cell clusters were annotated based on canonical gene and protein markers of various immune cell types. To evaluate potential cell type proportion differences between cGvHD and non-cGvHD patients, we applied a logit transform to the cell type proportion and fitted the transformed proportions using multiple regression with cGvHD status as the variable of interest while accounting for batch and relapse. To evaluate the effect of relapse, we swapped the roles of cGvHD and relapse in the regression model. We declared significance at an α of 0.05 with FDR correction for all combinations of cell types and group contrasts.

To identify differentially expressed genes between cGvHD and non-cGvHD patients for each cell type, we first applied a negative binomial mixed model, NEBULA [54], with gene expression counts of cells within a given cell type as the response, cGvHD status as the variable of interest, batch and relapse status as confounding factors, and within-subject correlation modeled by a random effect. Due to the empirical observation that mixed models applied to scRNAseq data might display an inflated false discovery rate [55], we further performed pseudobulk analysis. We generated a pseudobulk expression matrix for each cell type by applying “AggregateExpression” and performed multiple regression with normalized pseudobulk expression as the response, cGvHD status as the variable of interest, and batch and relapse status as confounding factors. We declared a gene as cGvHD differential if: (1) its *p*-value based on NEBULA passes FDR correction for all combinations of genes, cell types, and group contrasts, (2) its *p*-value based on pseudobulk analysis is <0.05, and (3) the directions of effect based on NEBULA and pseudobulk are the same. We employed this analysis approach due to our small sample size, which makes directly applying pseudobulk analysis too conservative. To identify relapse differential genes, we swapped the roles of cGvHD and relapse status in the above analysis. We applied the same analysis approach for identifying cGvHD and relapse differential proteins. To aid interpretation, we evaluated the overlap between the differentially expressed genes against GO genesets [56] using Fisher’s exact test. Due to the low number of genes measured by the immune-targeted AbSeq panel, we highlighted geneset enrichment at a nominal *p*-value of 0.05. We could not perform this geneset enrichment analysis for proteins, since only 41 proteins were measured.

Considering that some clinical factors (Table 1 and Table 2) could affect the associations between genes/proteins and cGvHD/relapse, we repeated the NEBULA analysis with clinical factors modeled as confounders. Due to the small sample size, we added each clinical factor into the NEBULA model one by one and correlated across all genes/proteins the signed *p*-values with and without accounting for the given clinical factor. We performed this robustness analysis only for clinical factors with adequate variation across patients, namely >3 patients who were different from others (Table 1 and Table 2). Examined factors included donor source, HLA matching, use of PTCy, ALL risk as well as aGvHD, given its relevance to cGvHD and relapse.

## 5. Conclusions

The results from this exploratory study suggest that there may be checkpoint inhibitor targets that are not upregulated in cGvHD at day 100. Moreover, the inhibitors of the IRE-1α/XBP-1 pathway, which have been shown in preclinical models to prevent cGvHD while preserving the GvL effect [57], may be targetable in high-risk patients to prevent later development of cGvHD, a marker not yet identified by examining peripheral blood. This exploratory study shows promising data that suggest we can separate the GvL response from the cGvHD and identify targetable interventions to minimize cGvHD and augment GvL in ALL HSCT in the future. However, the small sample size and the heterogeneity across patients warrant validation in larger, more uniform pediatric ALL HSCT cohorts.

## Figures and Tables

**Table 1:** Patient demographic and clinical characteristics.

UPN	BM Acquired Post-HSCT (Days)	Age at HSCT (Years)	Conditional Regiment	ALL Risk Factors	ATG or Other Sero-Therapy	Donor	Stem Cell Source	HLA Matching	GvHD Prophylaxis	CMV Serostatus (Recipient/ Donor)	ABO Mismatch (Recipient/ Donor)	Duration of Follow-Up (Months)
1	100	19	Flu/Tre/ Thiotepa	High risk Hypodiploid, CR2 Li-Fraumeni syndrome	None	Unrelated	Marrow	10/10	Tacro/MMF	Pos/Pos	B+/A+	14
2	100	9	TBI/Cy	High risk CR2	None	Related	Marrow	5/10	Tacro/MMF/PTCy	Pos/Pos	AB+/A+	10
3	100	16	Flu/TBI/Cy	post second HSCT, CR3	None	Related sibling	Marrow	5/10	Tacro/MMF/PTCy	Pos/Neg	A+/B+	18
4	100	18	Thiotepa/ TBI/Cy	High risk CR2	None	Related sibling	Marrow	9/10	Tacro/MMF	Neg/Pos	A+/A−	24
5	100	5	Etoposide/ TBI/Cy	High risk Refractory	None	Related sibling	Marrow	10/10	CsA/MMF	Neg/Neg	A+/A+	3, then moved
6	100	7	TBI/Cy	Standard risk CR2	None	Related sibling	Marrow	10/10	Tacro/MMF	Pos/Pos	O+/B+	20
7	60	8	TBI/Cy	Standard risk, CR3	None	Related sibling	Marrow	5/10	Tacro/MMF/PTCy	Pos/Neg	O+/A−	13
8	60	12	TBI/Cy	Standard risk, CR3	None	Related sibling	Marrow	5/10	Tacro/MMF/PTCy	Pos/Pos	O+/O+	4
9	100	17	Thiotepa/ TBI/Cy	High risk, CR2	None	Unrelated	Marrow	10/10	Tacro/Mtx	Neg/Neg	A+/O+	40
10	100	13	TBI/Cy	Standard risk, CR2	None	Unrelated	Marrow	10/10	Tacro/MMF	Neg/Neg	O+/O+	24
11	100	7	TBI/Cy	High Risk (Ph+), CR1	None	Related (father)	Marrow	10/10	Mtx	Pos/Neg	B+/O+	62
12	100	20	TBI/Cy	High Risk (Ph+), CR2	None	Related (brother)	Marrow	5/10	Tacro/MMF	Pos/Neg	A+/A+	7, then moved
13	100	15	TBI/Cy	High risk, CR2	None	Related (father)	Marrow	5/10	Tacro/MMF/PTCy	Pos/Pos	O+/O+	30
14	100	3	TBI/Cy	Standard risk, CR2	None	Related (father)	Marrow	5/10	Tacro/MMF/PTCy	Pos/Neg	A+/A+	24

**Table 2:** ALL relapse and cGvHD after HSCT.

UPN	Acute GvHD	aGvHD Grade	aGvHD Therapy	IST at Sampling	Chronic GvHD	Relapse	Outcome at 1 Year Follow-Up
1	Yes, GI	Grade 3	MP	None	Yes, oral	No	Alive
2	Yes, skin/GI	Grade 3	MP	None	Yes,	Yes	Alive
3	Yes, skin	Grade 1	Topical steroids	None	No	Yes	Alive
4	Yes, skin/GI	Grade 2	MP	None	No	Yes	Alive
5	Yes, skin	Grade 1	Topical steroids	None	No	No	Alive
6	No	No	No	None	No	Yes	Alive
7	No	No	No	None	Yes, lung	No	Alive
8	Yes, GI	Grade 2	MP/Ruxolitinib	MP/Ruxolitinib	Yes, renal	No	Dead
9	No	No	No	None	Yes, skin/oral mucosa	No	Alive
10	Yes, skin/liver	Grade 3	MP/Ruxolitinib	Yes	Yes, skin/liver	No	Alive
11	Yes, skin	Grade 3	MP	None	No	Yes	Alive
12	Yes, skin	Grade 1	Topical steroids	None	No	No	Alive
13	Yes, skin	Grade 3	Topical steroids/Tacro	None	No	No	Alive
14	Yes, skin	Grade 1	Topical Tacro	None	No	No	Alive

## Data Availability

The original contributions presented in this study are included in the article/Supplementary Materials. Further inquiries can be directed to the corresponding author.

## Supplementary Materials

The following supporting information can be downloaded at: https://www.mdpi.com/article/10.3390/ijms27052338/s1.
