# Supplementary material for: Early Marrow Microenvironment Immune Patterns After Hematopoietic Stem Cell Transplant in Pediatric Acute Lymphoblastic Leukemia Are Associated with Later Development of Chronic GvHD and Relapse
Source: Int J Mol Sci. 2026 Mar 2;27(5):2338. doi: 10.3390/ijms27052338 (PMC12985105; doi:10.3390/ijms27052338)
Supplement: Supplementary file 1 [file ijms-27-02338-s001.zip › ijms-4089933-supplementary.pdf]

Supplementary Table S1
List of 30 surface antibodies in the BD AbSeq Immune Discovery Panel (catalog #625970). The table includes antibody target, clone, and barcode information.

Specificity	Clone	Oligo ID
CD3	UCHT1	AHS0231
CD4	SK3	AHS0032
CD8	SK1	AHS0228
CD11c	B-Ly6	AHS0056
CD14	MPHIP9	AHS0037
CD16	3G8	AHS0053
CD19	SJ25C1	AHS0030
CD25	2A3	AHS0026
CD27	M-T271	AHS0025
CD28	L293	AHS0138
CD45RA	HI100	AHS0009
CD56	NCAM16	AHS0019
CD62L	DREG-56	AHS0049
CD127	HIL-7R-M21	AHS0028
CD134	ACT35	AHS0013
CD137	4B4-1	AHS0003
CD196 (CCR6)	11A9	AHS0034
CD197 (CCR7)	2-L1-A	AHS0273
CD272	J168-540	AHS0052
CD278	DX29	AHS0012
CD279	EH12.1	AHS0014
CD357 (GITR)	V27-580	AHS0104
CD366 (TIM-3)	7D3	AHS0016
HLA-DR	G46-6	AHS0035
IgD	IA6-2	AHS0058
IgM	G20-127	AHS0198
CD196 (CCR6)	11A9	AHS0034
CD197 (CCR7)	2-L1-A	AHS0273
CD272	J168-540	AHS0052
CD278	DX29	AHS0012
